# Hemostatic completion of percutaneous nephrolithotomy using electrocauterization and a clear amplatz renal sheath

**DOI:** 10.1590/S1677-5538.IBJU.2015.0434

**Published:** 2016

**Authors:** Ho Song Yu, Ji Won Ryu, Sun-Ouck Kim, Taek Won Kang, Dong Deuk Kwon, Kwangsung Park, Kyung Jin Oh

**Affiliations:** 1 Department of Urology, Chonnam National University Medical School, Gwangju, Republic of Korea

## Abstract

**Background and Purpose:**

A tubeless PCNL can reduce postoperative pain, the need for analgesics, hospital stay, and postoperative urinary leakage. However, perioperative or delayed bleeding remains the primary postoperative concern. We demonstrate a simple and cost-effective method to develop a clear nephrostomy tract after completion of a tubeless PCNL.

**Materials and Methods:**

Four consecutive patients with renal calculi >3cm underwent a tubeless PCNL. We used a 24 Fr nephroscope and a 24 Fr transurethral resectoscope. Intraoperative urologist-directed percutaneous renal access was performed under fluoroscopy. After calculi removal, active bleeders were identified via a clear Amplatz renal sheath. The sheath provided excellent visualization of the nephrostomy tract for the detection of bleeders and surrounding structures. Bleeders were electrocauterized using a roller barrel electrode. During extraction of the renal sheath, the surgeon can confirm hemostasis in the tract and apply intermittent suction.

**Results:**

Bleeding primarily originated from the torn calyeceal mucosa and the parenchyma. Tract electrocauterization was successful. All patients had mild hematuria, which resolved within two days. The average hemoglobin decrease was 1.65g/dL (0.8-2.1) and no patients required a transfusion. No perioperative complications occurred. On postoperative day 2, the patients could ambulate without a Foley catheter. During three months of follow-up, delayed bleeding or percutaneous urine leakage did not occur.

**Conclusions:**

Electrocauterization with a roller barrel electrode and a clear Amplatz renal sheath is an effective method to obtain hemostasis after completion of a PCNL. Our technique is cost-effective and readily adapted without the need for additional instruments.

## ARTICLE INFO

Available at: http://www.intbrazjurol.com.br/video-section/video-library/yu_170_171/

Int Braz J Urol. 2016; 42 (Video #2): 170-1

