# Supplementary material for: Hemostatic completion of percutaneous nephrolithotomy using electrocauterization and a clear amplatz renal sheath
Source: Int Braz J Urol. 2016 Jan-Feb;42(1):170–1. doi: 10.1590/S1677-5538.IBJU.2015.0434 (PMC4811245; doi:10.1590/S1677-5538.IBJU.2015.0434)
Supplement: Related texts

# Reply: EDITORIAL COMMENT

In the present video submission by Yu et al., the authors nicely depict a novel way bleeding encountered at the time of a percutaneous nephrolithotomy can be controlled using electrocautery and a clear Amplatz sheath. This is clearly one of the most challenging clinical scenarios encountered at time of such procedures can be addressed minimizing perioperative morbidity and the necessity for possible additional ancillary procedures. Developing innovative solutions to manage such surgical complications are strongly encouraged and I congratulate the authors in doing so particularly as this requires readily available surgical equipment at our disposition.

*Philippe E. Spiess, MD* *Associate Member, Department of Genitourinary Oncology* *Moffitt Cancer Center* *Tampa, FL, USA* *Video Section Editor,* *International Brazilian Journal of Urology* *E-mail: philippe.spiess@moffitt.org*
